# Privacy Preserving *k*-Nearest Neighbor for Medical Diagnosis in e-Health Cloud

**DOI:** 10.1155/2018/4073103

**Published:** 2018-10-15

**Authors:** Jeongsu Park, Dong Hoon Lee

**Affiliations:** Graduate School of Information Security, Korea University, Seoul, Republic of Korea

## Abstract

Cloud computing is highly suitable for medical diagnosis in e-health services where strong computing ability is required. However, in spite of the huge benefits of adopting the cloud computing, the medical diagnosis field is not yet ready to adopt the cloud computing because it contains sensitive data and hence using the cloud computing might cause a great concern in privacy infringement. For instance, a compromised e-health cloud server might expose the medical dataset outsourced from multiple medical data owners or infringe on the privacy of a patient inquirer by leaking his/her symptom or diagnosis result. In this paper, we propose a medical diagnosis system using e-health cloud servers in a privacy preserving manner when medical datasets are owned by multiple data owners. The proposed system is the first one that achieves the privacy of medical dataset, symptoms, and diagnosis results and hides the data access pattern even from e-health cloud servers performing computations using the data while it is still robust against collusion of the entities. As a building block of the proposed diagnosis system, we design a novel privacy preserving protocol for finding the *k* data with the highest similarity (PE-FTK) to a given symptom. The protocol reduces the average running time by 35% compared to that of a previous work in the literature. Moreover, the result of the previous work is probabilistic, i.e., the result can contain some error, while the result of our PE-FTK is deterministic, i.e., the result is correct without any error probability.

## 1. Introduction

Cloud computing, as an emerging computing paradigm, is revolutionizing the data processing methodology of many organizations because of its resource efficiency and reduction in management cost. As the costs of healthcare services rise, e-health is considered as one of the promising fields that could benefit from using cloud computing [1, 2]. Among various health services, medical diagnosis is especially well suited for the e-health cloud, because the diagnosis requires heavy computational ability and can be implemented on a pay-as-you-use model on the Internet.

Meanwhile, adopting cloud computing for medical diagnosis causes privacy issues because of the sensitive personal information contained in medical data. Specifically, if medical data owners such as hospitals outsource their medical diagnosis dataset in the open to e-health cloud, a compromised e-health cloud service provider might expose them. Similarly, if a patient inquirer sends and receives his/her symptom and diagnosis result in the open with the e-health cloud for diagnosis service, the compromised e-health cloud service provider might infringe on his/her privacy by exposing them. Even though the medical data owners and the patient inquirer encrypt them before sending them to the e-health cloud to protect their privacy, it is still possible that the compromised e-health cloud service provider might obtain additional information by observing data access patterns during processing.

The Health Insurance Portability and Accountability Act (HIPAA) regulates the privacy and security of individually identifiable health information to be guaranteed obligatorily [3]. The privacy and security regulations of HIPAA were improved in the Health Information Technology for Economic and Clinical Health (HITECH) Act [4]. Unfortunately, these acts do not suggest the technical methods for the privacy.

For medical diagnosis, case-based reasoning (CBR), which has been applied to the medical diagnosis since late 1980 [5], is a well-established problem-solving methodology. Given a problem (i.e., symptom), CBR provides its solution (i.e., diagnosis result) by referencing the cases with the most similar problem to the given problem among previous ones (i.e., medical diagnosis dataset) in case library where a case consists of a problem and its solution [6, 7]. One of the most important functionalities in CBR is to find the most similar cases to provide the solution to a given problem. For this purpose, many papers and systems [7–11] adopted *k*-nearest neighbor (*k*NN) classification. In other words, *k*NN classification for a query selects the *k* most similar data to the query in a classified dataset and determines the class of the query as the majority class of the *k* selected data [12]. It is fairly simple, has great performance, and gives a quite good result.

In real healthcare service environment, health records are owned by multiple data owners such as hospitals, which are unwilling to reveal the health records due to privacy or legal issue. If a data owner collects the health records to outsource them to e-health cloud servers, it brings privacy concerns. Unfortunately, most of the previous works to compute *k*NN in privacy preserving manner assumed that there exists only one data owner rather than multiple data owners [13–19].

### 1.1. Contribution

The main theme of this paper is to design a privacy preserving *k*NN classification, so-called PP*k*NN [15], with multiple data owners for medical diagnosis. For privacy, we provide the privacy of medical dataset outsourced by multiple dataset owners, a symptom of patient inquirer, data access patterns during computation, and diagnosis results as PP*k*NN result. For security, we provide robustness against collusion among cloud servers, collusion between any data owner and cloud server, or collusion between inquirer and cloud server. There have been some results on PP*k*NN using cloud computing with multiple data owners. The authors of [20] proposed a privacy preserving kernel density estimation instead of PP*k*NN and demonstrated that the result accuracy is similar to that of PP*k*NN in many applications. They also introduced various realistic threats which can occur in the multiple data owner environment and discussed privacy of PP*k*NN classification. But, their protocol does not consider the privacy of the *k*NN result and data access pattern. In the work of [21], its PP*k*NN provides the privacy of dataset, input query, *k*NN result, and data access pattern. But it is vulnerable to collusion attacks. In other words, it assumed that there is neither collusion among cloud servers nor collusion between any data owner and cloud server. We summarize functionalities provided in the previous works and our PP*k*NN in Table 1.

As one of the building blocks of our PP*k*NN, we propose the improved method to find *k* data with the highest similarity (PE-FTK). It reduces the average running time by 35% when compared to the previous work [22]. The number of rounds and the running time increases little as the number of data or *k* increases. Moreover, the result of the previous work is probabilistic, i.e., its result can contain some error, while the proposed PE-FTK is deterministic, i.e., our result is correct without any error probability. Thus, our PE-FTK is more suitable for medical diagnosis to handle sensitive medical data. We stress that our work is meaningful in terms of privacy preserving and efficient protocol to find *k* data with the highest value (top-*k* data) using cloud computing.

As mentioned in [23], privacy preserving cloud computing with multiple data owners and inquirers (they denoted that model as stateful private multi-client computing) cannot be realized with a single cloud server using only cryptography, and adopting distributed multiple cloud servers can be an alternative. We thus realize our PP*k*NN using multiparty computation (MPC) based on secret sharing to compute *k*NN result in distributed manner without any trusted server.

In MPC based on secret sharing, data are to be shared among multiple cloud servers and each share reveals nothing on the original data, which can be reconstructed only when a sufficient number (i.e., more than the predefined threshold value) of shares are combined together. Since our PP*k*NN is designed using MPC, it is robust to collusion attack. In other words, it allows for an adversary to compromise some of e-health cloud servers. The allowed number of the compromised cloud servers depends on the MPC protocol to be adopted. For instance, when GMW protocol [24] is applied, our PP*k*NN can compute *k*NN results in the privacy preserving manner even if an adversary compromises all e-health cloud servers except one.

The remaining part of this paper is organized as follows: in Section 2, we explain MPC primitive, complexity, and *k*NN as preliminaries and then outline the proposed PP*k*NN and attack scenarios in Section 3. In Section 4, we present the proposed PE-FTK as main contribution and then present the proposed PP*k*NN. In Section 5, we analyze the efficiency and discuss the security of PE-FTK and PP*k*NN. In Section 6, we review the previous works related to PP*k*NN and privacy preserving top-*k* protocol and lastly conclude this paper in Section 7.

## 2. Preliminaries

We explain MPC protocols based on Shamir's secret sharing in Section 2.1 and Section 2.2 by which our proposed protocols are constructed (we implemented our PE-FTK using the source code opened in the previous work [25] which is the MPC protocol based on Shamir's secret sharing). However, since the proposed protocols can be constructed by not only MPC based on Shamir's secret sharing but also those based on other secret sharing, such as [24], we consider MPC applying to our proposed protocol as those based on secret sharing throughout this paper.

### 2.1. Multiparty Computation Based on Shamir's Secret Sharing

MPC allows a set of parties (i.e., cloud servers) to jointly compute an agreed function on their inputs in a distributed fashion and to obtain the results of the function but nothing else. Each party receives shares generated from input values of function and computes results using the shares. MPC assumes that it allows for an adversary to compromise at most *t* parties, and their *t* shares do not involve any information on the original data. In other words, since any adversary to compromise at most *t* parties does not obtain information on the original data, MPC allows for parties to carry out secure computation without a trusted third party.

MPC based on secret sharing proceeds in three phases: input sharing, computation, and output reconstruction. In the input sharing phase, a party or an external entity holding a secret *s* generates a random polynomial *f*_*s*_(*x*) of degree *t* at most with *f*_*s*_(0) = *s* where *t* is the number of corrupted parties and sends its share *f*_*s*_(*α*_*i*_) to each party *P*_*i*_ where *α*_*i*_ is any distinct nonzero element. In this paper, we denote the shares by [*s*]=(*f*_*s*_(*α*_1_),…, *f*_*s*_(*α*_*n*_)) where *n* is the number of parties. In the computation phase, parties carry out a protocol according to a gate in circuit realizing the function agreed by the parties in advance and obtain result in shared representation. Lastly, in the output reconstruction phase, the parties send their own computed shares to the other parties and then reconstruct the final result from the received shares. Bitwise sharing shares a secret *s* in bitwise shared representation, i.e., the bitwise share is [*s*]_*B*_={[*s*_*l*−1_],…, [*s*_0_]} where *s*=∑_*i*=0_^*l*−1^2^*i*^*s*_*i*_, *s*_*i*_ ∈ {0, 1} and *l* is the size of the secret *s*.

### 2.2. Addition and Multiplication of Multiparty Computation

Since Shamir's secret sharing has a linear property, addition in MPC is homomorphic. For addition of [*a*] and [*b*], each party *P*_*i*_ locally adds up its own shares *f*_*a*_(*α*_*i*_) and *f*_*b*_(*α*_*i*_) without any communication. We denote the addition of MPC by [*a*] + [*b*] = [*a* + *b*]. Similarly, since multiplication by a public constant *c* is also homomorphic, each party *P*_*i*_ holding share *f*_*a*_(*α*_*i*_) locally multiplies *f*_*a*_(*α*_*i*_) by the public constant *c* without any communication. We denote multiplication by a public constant *c* by *c*·[*a*] = [*c*·*a*].

Multiplication by two shares [*a*] and [*b*] requires communication once. Specifically, each party *P*_*i*_ locally multiplies its own shares *f*_*a*_(*α*_*i*_) and *f*_*b*_(*α*_*i*_), i.e., *h*_*i*_=*f*_*a*_(*α*_*i*_) × *f*_*b*_(*α*_*i*_), generates shares of the *h*_*i*_ by Shamir's secret sharing, i.e., [*h*_*i*_]=(*f*_*h*_*i*__(*α*_1_),…, *f*_*h*_*i*__(*α*_*n*_)), for *i *= 1,…, *n* (*n* is the number of parties), and sends the share *f*_*h*_*i*__(*α*_*j*_) to other party *P*_*j*_. Lastly, each party *P*_*i*_ computes ∑_*j*_*λ*_*j*_*f*_*h*_*j*__(*α*_*i*_), where *λ*_*j*_ is the recombination vector and public information that all parties can compute. For more details, refer to [26]. The circuit randomization method [27] enables to locally perform multiplication without any communication by using precomputed random shares [*x*], [*y*], and [*z*] where [*z*] = [*xy*].

### 2.3. Comparison and Equality

Our proposed protocol uses comparison (lessThan) and equality MPC operations as well as basic addition and multiplication. In [25], comparison MPC operation requires 24*l* + 5 multiplications in 2*l* + 10 rounds, and the equality MPC operation requires *l* + 1 multiplications in *l* rounds (we implemented the proposed PE-FTK using the library of [28] to implement the comparison and equality operations proposed in [25]. Their running time is optimized by reducing the number of multiplications although their round complexity is linear in the length of data. For more details, refer to [25]), where *l* is the size of data.  Table 2 shows notations for MPC operations used in our protocol. The comparison and equality MPC operations are proved formally in the previous works, [29, 30] and therefore, we skip a formal proof in this paper.

### 2.4. Complexity

We evaluate the efficiency of a protocol in terms of both the number of rounds and the amount of communication. We measure the round complexity by the invocation count of a dominant operation performed in parallel and the communication complexity by the total number of invocations of the dominant operation to be carried out, as in [29, 30]. In other words, the round complexity denotes the time required to complete a protocol, and communication complexity denotes the amount of data sent and received in a protocol.

### 2.5. *k*-Nearest Neighbor

*k*NN classification [31], as an instance-based learning algorithm, is one of the simplest and oldest nonparametric pattern classification techniques and results in a competitive outcome. It selects *k* data most similar to an unclassified input query (i.e., input symptom) in classified dataset (i.e., medical dataset) and classifies the input query into the class (i.e., diagnosis result) with the majority class of the selected *k* data. Its performance depends on similarity computation. Many papers and medical diagnosis systems related to *k*NN adopted Euclidean distance for a similarity measure [6].

## 3. Overview

We outline the proposed PP*k*NN in Section 3.1 and explain how to generate global dataset in shared representation from horizontally or vertically distributed datasets of multiple data owners for input of PP*k*NN or PE-FTK in Section 3.2. Then, we explain attack scenarios in Section 3.3.

### 3.1. System Model

**Figure 1:**
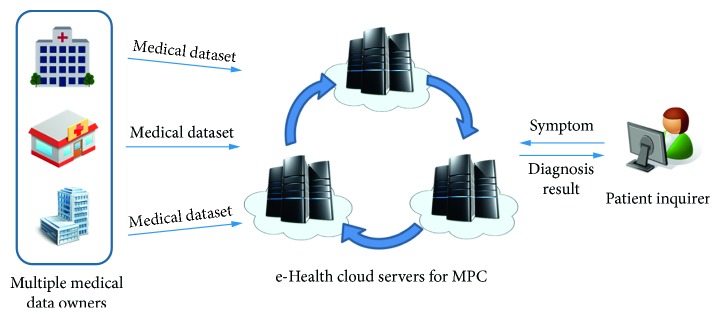
Architecture of the proposed PP*k*NN system for medical diagnosis.

The proposed PP*k*NN consists of multiple medical data owners, e-health cloud servers, and a patient inquirer as shown in Figure 1. Organizations such as hospitals holding medical diagnosis datasets can be medical data owners. For medical diagnosis service, multiple medical data owners outsource their medical datasets to e-health cloud servers to utilize their huge computing resources and benefit from their management cost. A patient inquirer wishing to have a medical examination sends his/her symptom to the e-health cloud servers. The e-health cloud servers carry out PP*k*NN classification as a part of the medical diagnosis and return the result back to the patient inquirer. We assume that the entities are connected on a secured and authenticated channel. This means that an adversary cannot eavesdrop on the communication between the entities.

We represent the medical data by symptom and its diagnosis result, denoted by ($\vec{d_{i}}$, *c*_*i*_). We assume that the symptom consists of *m* details, denoted by *m*-dimensional vector $\vec{d_{i}}$ = (*d*_*i*,1_,…, *d*_*i,m*_), and the diagnosis result *c*_*i*_ is in {1,…, *v*}, represented as *v*-bit value. If the diagnosis result *c*_*i*_ is *α* ∈{1,…, *v*}, the only *α*-th bit is 1 and the other bits are all 0.

We assume that the input symptom of a patient inquirer consists of *m* details and denote it by *m*-dimensional vector $\vec{q}$ = (*q*_*1*_,…, *q*_*m*_) as the symptom of the medical data. We also assume that the result sent from e-health cloud servers is in {1,…, *v*}. We denote it by $\vec{scr}$ = (*scr*_1_,…, *scr*_*v*_), where *scr*_*i*_ is the score of each disease. The diagnosis result for the symptom of patient inquirer is the disease with the highest score.

### 3.2. Generating an Input Dataset from Horizontally or Vertically Distributed Data

In this subsection, we explain how cloud servers privately generate global dataset from datasets distributed to multiple data owners for PP*k*NN or PE-FTK. The data distribution approach is classified as horizontally distributed dataset and vertically distributed dataset [32]. In the horizontally distributed dataset, each data owner holds some records of global dataset which have the same set of attributes. In the vertically distributed dataset, each data owner holds data corresponding to some attributes of global dataset.

In order to carry out the proposed PP*k*NN or PE-FTK on global dataset of multiple data owners, they carry out the input sharing phase by sending shares generated from their datasets to each cloud server as described in Section 2.1. For instance, in the horizontally distributed dataset, if a data owner A stores {(*d*_1_, *e*_1_),…, (*d*_*n*_A__, *e*_*n*_A__)} and a data owner B stores {(*d*_*n*_A_+1_, *e*_*n*_A_+1_),…, (*d*_*n*_A_+*n*_B__, *e*_*n*_A_+*n*_B__)}, the global dataset which cloud servers store after input sharing phase is {([*d*_1_], [*e*_1_]),…, ([*d*_*n*_], [*e*_*n*_])} for *n*=*n*_A_+*n*_B_. In the vertically distributed dataset, if a data owner *A* stores {*d*_1_,…, *d*_*n*_} and a data owner *B* stores {*e*_1_,…, *e*_*n*_}, the global dataset is also {([*d*_1_], [*e*_1_]),…, ([*d*_*n*_], [*e*_*n*_])}. In order to generate one column dataset for PE-FTK, cloud servers additionally carry out computation (e.g., *computeSimilarity* in this paper) and generate the global dataset {*f*([*d*_1_], [*e*_1_]),…, *f*([*d*_*n*_], [*e*_*n*_])} as input dataset.

### 3.3. Attack Scenarios

We consider a semi-honest adversary model where a compromised entity follows a specified protocol but tries to obtain additional information on dataset of data owners, input query, intermediate results, and *k*NN result during the protocol. Our PP*k*NN allows for an adversary to compromise any entity, and we also consider multidata owner outsourced model defined in [20] where an adversary can compromise several entities simultaneously and carry out collusion attack. However the authors of [20] showed that the adversary, which compromises both data owners and inquirer and performs collusion attack, can obtain additional information on dataset of data owner regardless of protocol design or encryption scheme, even if cloud servers store the dataset in encrypted form. Therefore, we exclude the attack to compromise both data owners and inquirer and consider the remaining attacks. In other words, we consider the attacks where an adversary compromises cloud servers and data owners, cloud servers and inquirer, and each entity.

The attack scenarios in our PP*k*NN are as follows: a data owner tries to obtain information on dataset of another data owner. An inquirer also tries to obtain information on dataset stored in cloud servers by analyzing input query and *k*NN result occurred in communication with the cloud servers. Cloud servers try to obtain information on the internally stored dataset, the input query sent from an inquirer, intermediate results, and *k*NN result. Furthermore, since the compromised cloud servers can also collude with data owners or inquirer in a multidata owner outsourced model [20] (we assume that it allows for an adversary to compromise at most *t* entities including data owner or inquirer), they try to obtain information from their own randomized dataset in the way that they send an input query via the compromised inquirer to themselves and observe data access patterns during computation. With the information from the attack scenario, they can obtain information on input query sent by another inquirer.

Since our PP*k*NN is constructed with MPC, it allows for an adversary to compromise some of cloud servers. The proposed PP*k*NN can be realized by applying MPC based on secret sharing according to the number of cloud servers and the expected compromised cloud servers among them. Even though we consider semi-honest adversary model in our work, it is possible to realize the protocols of cloud servers secure against malicious adversary if we apply MPC secure against malicious adversary to the proposed protocol of cloud servers.

#### 3.3.1. Notations

For simplicity, 〚*n*〛 means a set {1, 2,…, *n*}. For a set A={*i*_1_, *i*_2_,…, *i*_*k*_}, {*d*_*i*_}_*i*∈A_ means {*d*_*i*_1__, *d*_*i*_2__,…, *d*_*i*_*k*__}.

## 4. Proposed Protocols

PP*k*NN firstly computes similarities between input query and each data in dataset (*computeSimilarity*), converts the similarities in bitwise shared representation (*Bit-Decomposition*), and selects *k* data with the highest similarities (PE-FTK). Among the subprotocols, we focus on the most important PE-FTK to select top-*k* similarities and present it in Section 4.1, and the other subprotocols utilize the previous works. We construct the proposed PP*k*NN using the subprotocols in Section 4.2.

### 4.1. Privacy Preserving and Efficient Protocol to Find the Top-*K* Data (PE-FTK)

The basic idea of PE-FTK is to find the top-*k* data according to the arrangement of bitwise 1. Specifically, the higher value out of two values denotes that, when examining and comparing each bit of the two values from the most significant bit to the least significant bit, bitwise 1 appears earlier in the higher value than in the lower value. For example, when comparing two 4-bit-data 4 and 3 (0100 and 0011 in binary), since the second bit (from the most significant bit) of data 4 is 1 while the second bit of data 3 is 0, the data 4 is higher. As another example, when comparing two 4-bit-data 6 and 5 (0110 and 0101 in binary), since the second bit of both data is 1 but the third bit of data 6 is 1 while the third bit of data 5 is 0, the data 6 is higher.

While PE-FTK examines each bit of all data from the most significant bit (we will call it *bit-round*), it counts the number of data whose current bit is 1, i.e., it adds up the current bits of all data, since a bit is 0 or 1. Then, it adds the count and the number of data in which bitwise 1 already appears in a prior bit, i.e., the result dataset in prior bit-round, and compares the sum with *k*. The detailed procedure is as follows:

1. While examining each bit from the most significant bit to the least significant bit, PE-FTK computes *Cnt* by adding the sum of the current bits of data in which bitwise 0 continually appears in prior bit and the number of data in which bitwise 1 appears in prior bit, i.e., the result dataset in prior bit-round, and compares the *Cnt* with *k.*
2. *Cnt* > *k*: it carries out step (3).
3. *Cnt* == *k*: it contains in the result dataset, the data whose current bit value is 1. Then, it outputs the result dataset and terminates.
4. *Cnt* < *k*: it includes in the result dataset, the data whose current bit value is 1 and repeats step (1).
5. It decides candidate data, that is, the data whose current bit is 1 among the data in which bitwise 0 continually appears in the prior bit
6. For the next bit of candidate data, it computes *Cnt* by adding the sum of the current bits of the candidate data and the number of result dataset in prior bit-round and compares the *Cnt* with *k.*
7. *Cnt* == *k*: it contains in the result dataset the candidate data whose current bit value is 1. Then, it outputs the result dataset and terminates.
8. *Cnt* > *k*: it removes the candidate data whose current bit value is 0 from them and carries out step (4).
9. *Cnt* < *k*: it includes in the result dataset, the candidate data whose current bit value is 1, and then it carries out step (4).

Table 3 and 4 shows an example of PE-FTK where a dataset is {16, 12, 11, 10, 9} and *k *= 3, and thus the result dataset is {16, 12, 11}. We define PE-FTK as Algorithm 1. Recall that bitwise share is [*s*_*i*_]_*B*_={[*s*_*i*,*j*_]}_*j*∈{*l* − 1, ⋯,0}_ where *s*_*i*_=∑_*j*=0_^*l*−1^ 2^*j*^*s*_*i*,*j*_, *s*_*i*,*j*_ ∈ {0, 1} and *l* is the size of a secret *s*_*i*_.

PE-FTK consists of part 1 (lines 2–13) and part 2 (lines 15–24). When it checks the *j*-th bit in part 1, it computes *Cnt* by adding the number of data in which bitwise 1 appears from the (*l* − 1)-th bit to the (*j *+ 1)-th bit and the number of data where the *j*-th bit is 1 among the data in which bitwise 0 continually appears from the (*l* − 1)-th bit to the (*j + *1)-th bit (line 3) and compares the *Cnt* with *k* (lines 6 and 10). In the case where *Cnt* is less than or equal to *k*, it includes in the result dataset, the data where the *j*-th bit is 1 (line 9), and in the case where *Cnt* is larger than *k*, it proceeds to part 2 (line 7). In part 2, it finds the top-*k* data among candidate data ([*Can*_*i*_]=[1]). It computes *Cnt* of the current bit in the same manner as the part 1 (line 16). In the case where *Cnt* is not equal to *k*, it computes the result dataset (line 22) and candidate dataset (line 23), respectively, and otherwise, it computes and returns the result dataset (lines 18-19).

### 4.2. Privacy Preserving *k*-Nearest Neighbor (PP*k*NN)

We present the PPkNN protocol in Algorithm 2. There are a variety of similarity measures for *computeSimilarity* protocol and we consider the squared Euclidean distance [33] in this paper. The *Bit-Decomposition* protocol decomposes a shared secret [s] into a bitwise shared secret [*s*]_*B*_={[*s*_*l*−1_],…, [*s*_0_]} where *s*_*i*_ ∈ {0, 1} for *i* = 0, *…*, *l* − 1. For an efficient bit-decomposition protocol, refer to [34].

PP*k*NN computes similarity between an input query $\left[\vec{q}\right]$ and each data $\left[\vec{d_{i}}\right]$ (line 1) and selects *k* data with the highest similarity (line 3). If the data $\left[\vec{d_{i}}\right]$ is one of the top-*k* data, it holds [*ck*_*i*,*j*_]=[*c*_*i*,*j*_] for *j* ∈ 〚*v*〛 since [*Res*_*i*_] = [1], and otherwise, [*ck*_*i,j*_] = [0] for *j* ∈ 〚*v*〛 since [*Res*_*i*_] = [0] (line 4). Thus, the value [*scr*_*j*_] to add up {[*ck*_*i*,*j*_]}_*i*∈〚*n*〛_ (line 5) is the score value to aggregate the *j*-th class of *k* data most similar to the query $\left[\vec{q}\right]$. Cloud servers send the result shares of $\left[\vec{scr}\right]={\left\{\left[scr_{j}\right]\right\}}_{j\in 〚v〛}$ to the inquirer, and it then reconstructs and obtains $\vec{scr}={\left\{scr_{j}\right\}}_{j\in 〚v〛}$. The class information with the highest value among {*scr*_*j*_}_*j*∈〚*v*〛_ is the class (i.e., diagnosis result) for input query $\vec{q}$ (i.e., input symptom) as the result of *k*NN classification.

## 5. Efficiency and Security

In this section, we discuss the efficiency and the security of the proposed protocols. Specifically, we analyze the empirical result of PE-FTK implementation in Section 5.1 and measure the complexity of PE-FTK and PP*k*NN in Section 5.2. We discuss the security of PE-FTK in Section 5.3 and that of PP*k*NN in Section 5.4.

### 5.1. Empirical Results of PE-FTK

We implemented the proposed PE-FTK with the source code of [28] based on Java which is opened in the previous work [25] and conducted experiments to confirm its performance. Specifically, we first experimented PE-FTK implementation for five cloud servers to find the top 100 data among 1000 data of 33 bits generated in random and then varied the number of data, length of data, and *k* where each experiment is conducted 30 times. Each cloud server was run on a separate server, and intermediate results across 100 Mb/s network were communicated. A cloud server used an Intel Core i7 2.4 GHz CPU.

**Figure 2:**
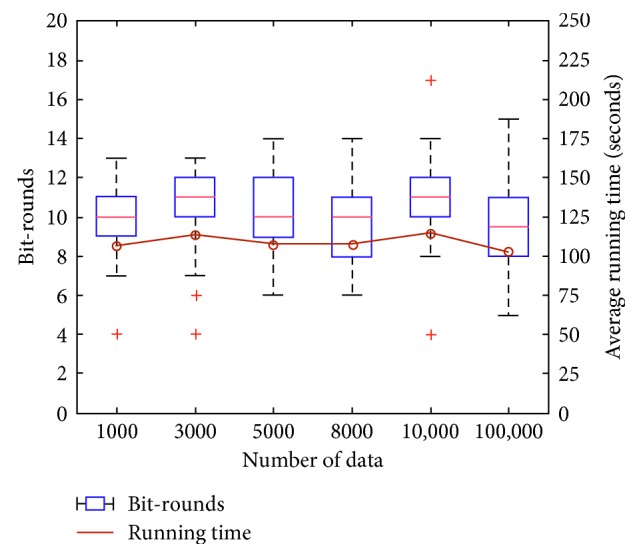
The number of bit-rounds and average running time according to the number of data.

**Figure 3:**
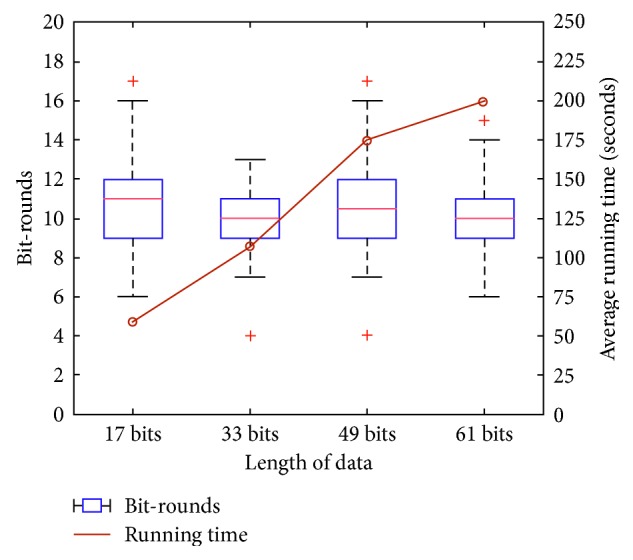
The number of bit-rounds and average running time according to the length of data.

**Figure 4:**
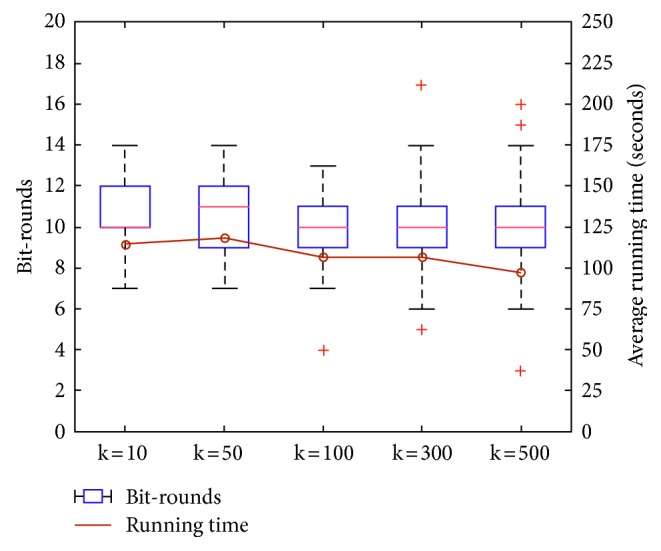
The number of bit-rounds and average running time according to *k*.

Figures 2–4 show the distribution of the number of bit-rounds and average running time using a box-and-whisker plot and line graph, respectively. In the box-and-whisker plot, the central mark and each edge of the box represent the median, the 8th (Q1) and the 23rd (Q3) of the number of bit-rounds, respectively. The whisker represents the range not to be considered, i.e., outliers, which means the range larger than Q3 + 1.5 (Q3−Q1) or smaller than Q1-1.5 (Q3-Q1) as [22].

As seen in PE-FTK (Algorithm 1), the computation cost of part 2 (lines 15-24) contributes most to the complexity of PE-FTK and that of part 1 (lines 2-13) is relatively low. In other words, the part 1 requires one round (one invocation) of multiplication each bit-round, while the part 2 requires the expensive comparison and equality operations once as well as 3 rounds (5 invocations) of multiplication each bit-round. According to the previous result [25] used to implement PE-FTK, the comparison operation requires 76 rounds (797 invocations) of multiplication and the equality operation requires 34 rounds (34 invocations) of multiplication in the case of 33-bit data. Therefore, the execution of part 2 is a dominant factor of the complexity of PE-FTK.

Table 5 shows our PE-FTK is more efficient than the previous work [22] (as the number of input parties increases in the previous work [22], the number of round increases, since the previous work runs the collision resolution phase to reduce global collision. However, the number of bit-rounds and the running time of our PE-FTK do not increase, since it outputs deterministic result) in terms of average running time for one round and total running time. This is because the previous work requires the expensive comparison operation one more each its round. Our experimental results show that the distribution of the number of bit-rounds and the average running time of PE-FTK little increase, even when the number of data, length of data, and *k* increase, except for the running time according to the length of data. We observed that our PE-FTK found the top-*k* data between 9.7 and 11.1 bit-rounds and took between 98.23 and 123.83 seconds for dataset generated at random. Moreover, the experimental results show a great variance because the data are at random.

Figure 2 shows that the number of bit-rounds and the average running time of PE-FTK do not increase in proportion to the increasing number of data. As seen in PE-FTK, the number of multiplication invocations is proportional to the number of data (*n* invocations in part 1 and 5*n* invocations in part 2, where *n* is the number of data), but they have little influence on running time since these multiplications can be carried out in parallel. Furthermore, since the expensive comparison and equality operations take *Cnt* and *k* unrelated to the number of data as input, the number of data does not have an influence on the number of bit-rounds and the average running time of PE-FTK.

Figure 3 shows that the number of bit-rounds does not increase as the length of data increases, but the average running time increases. The reason is not PE-FTK. It is because the comparison and equality in the library [25, 28] used to implement PE-FTK are linear in the length of data. In other words, since the complexities of the comparison and equality operations in the library [28] are linear in the length of data, the average running time of PE-FTK implementation increases in proportion to the length of data. Therefore, if we implement PE-FTK with the library in which the complexities of comparison and equality are constant [29, 30], the running time does not increase as the number of bit-rounds.

Figure 4 shows that the number of bit-rounds and the average running time of PE-FTK are unrelated to *k*. The round of the previous work [22] increases according to the increase of *k*, while PE-FTK does not require additional operations for the high value of *k*. In other words, since the number of expensive comparison and equality operations does not increase according to the increase of *k*, the value of *k* does not have an influence on the number of bit-rounds and the running time.

### 5.2. Complexity

As explained above, we evaluated the complexity of PE-FTK with the execution count of part 2 (lines 15–24), since the complexity of part 2 contributes most to that of PE-FTK.  Table 6 shows the complexity of PE-FTK in comparison to that of the previous work [22]. The previous work requires two rounds of comparison (*n* + 1 invocations) each its round, since it compares *τ* (the median of data bound) to all *n* data and the number of larger data to *k*. Since the previous work requires one more round of comparison (*n* invocations more) each its round, our PE-FTK is more efficient. In the experiment of PE-FTK with random data, the execution count *α* of part 1 was mostly one or two. However, if most data are the values smaller than 10 bits size, PE-FTK can be more efficient since the execution count of part 1 increases and that of part 2 decreases.

The complexity of PP*k*NN consists of executions of *computeSimilarity* (line 1), *Bit-Decomposition* (line 2), *PE-FTK* (line 3), and multiplications of line 4. Since we consider the squared Euclidean distance [33] to compute similarity, the *computeSimilarity* requires one round of multiplication (*nm* invocations). The *Bit-Decomposition*, which represents the similarity values in bitwise shared representation for PE-FTK, is known as a comparatively expensive operation. However, in state-of-the-art research [34], the author constructed a very efficient bit-decomposition protocol using precomputed random values. It requires (3*l* − 2*u*) multiplications in (*l/u* + 1) rounds where *l* is the length of data and *u* is the number of bits to convert in one round. For more details, refer to [34]. Lastly, line 4 requires one round of multiplication (*nv* invocations). Consequently, since the round complexity, which relates to the time to complete a protocol, is not proportional to the number of data which is quite large in most cases, our PP*k*NN is relatively efficient.

### 5.3. Security of PE-FTK

In the part 1 of our PE-FTK, cloud servers reconstruct the number of the highest data (*Cnt*) each bit-round for efficiency. In other words, until the number of the highest data is larger than *k* (part 1), the number of the highest data is leaked for each bit-round. However, it does not leak what data is the highest data and what the exact value of the highest data is. It leaks that bitwise 1 appears in current bit of *Cnt* data among all data. The information does not give an unreasonable amount of information on input dataset to cloud servers.

As a variation of PE-FTK, it is possible to find the top-*k* data without reconstructing *Cnt* in part 1. It requires comparison operation (line 6 in Algorithm 1) and equality operation (line 10 in Algorithm 1) once each bit-round, respectively. However, the previous work [22] requires *n* comparisons in one round each bit-round (totally, *nl* comparisons in *l* rounds) more in comparison to the variation where the comparison operation is the expensive operation in our proposed protocols, and thus the variation is still more efficient than the previous work. Moreover, since the number of comparison and equality operations is unrelated to the number of data, the length of data and *k*, even if they increase, the efficiency is similar to that of Section 5.1.

### 5.4. Security of PP*k*NN

We show that the proposed PP*k*NN is secure against the threats mentioned in Section 3.3. Specifically, we show that our PP*k*NN provides the privacy of dataset of data owners, input query, *k*NN result, and data access pattern for three attack scenarios to compromise each entity, cloud servers and data owners, and cloud servers and inquirer.

#### 5.4.1. Privacy of Dataset

Since data owners send randomized shares of their dataset to each of cloud servers in the input sharing phase, at most *t* compromised cloud servers cannot obtain any information on the original dataset from their shares as explained in Section 2.1. Similarly, since *t* compromised cloud servers can obtain at most *t* shares of the intermediate results during MPC processing, they cannot obtain any information on the intermediate results. Since data owners do not interact with other data owners and do not receive any data from other entities, the compromised data owners cannot obtain any information. Even if compromised cloud servers collude with data owner or inquirer, they obtain at most *t* shares of each dataset and thus it cannot obtain any information on dataset.

#### 5.4.2. Privacy of Input Query and *k*NN Result

Similar to data owners, an inquirer sends to each of cloud servers the randomized share of an input query generated in secret sharing phase and receives *k*NN result in shared representation from each of the cloud servers. Note that the *k*NN result is reconstructed to the inquirer rather than the cloud servers. Since the adversary can obtain at most *t* shares of the input query and the *k*NN result, it is impossible to leak their information.

#### 5.4.3. Privacy of Data Access Pattern

Compromised cloud servers can attempt to guess additional information by observing data access patterns even though the stored data are randomized. For example, when the compromised cloud servers collude with an inquirer, the compromised inquirer can send an input query to cloud servers and the compromised cloud servers can observe the data access patterns. However, since the cloud servers access all data to compute *k*NN result, the compromised cloud servers cannot guess the relation between the input query and the data access patterns.

## 6. Related Work

In this section, we review existing works related to PP*k*NN and a privacy preserving top-*k* protocol.

### 6.1. Privacy Preserving *k*-Nearest Neighbor Protocols

After Lindell and Pinkas first introduced privacy preserving data mining in [35], many researchers proposed PP*k*NN schemes. In [33], Shaneck et al. proposed the PP*k*NN algorithm over a horizontally distributed dataset, but it leaks some information. Qi et al. [36] resolved the information disclosure problem of [33] with a homomorphic encryption such as the Paillier cryptosystem, but their protocol also executes in a horizontally distributed data model. Further, Xiong et al. [37] proposed a PP*k*NN scheme which does not provide query privacy as its query is publicly known, and the protocol is also executed in a horizontally distributed data model.

In [13], Yao et al. relaxed the PP*k*NN requirement in which the protocol finds the partition containing the nearest neighbor for a query instead of the exact nearest neighbor. In their protocol, a data owner and inquirer must be trusted because they share a secret key. In [14], Elmehdwi et al. proposed a scheme using the Paillier cryptosystem with a homomorphic property, which provides both data and query privacy, and hides the access pattern. Then, they improved their work in [15] and formally proved the scheme that outputs the query class information in encrypted data. However, they did not consider the untrusting multiple data owner model. In [16], Zhu et al. proposed a PP*k*NN scheme in which a data owner does not expose the secret key to an inquirer but it encrypts the query by interacting with the inquirer. Hence, the data owner maintains the online connection for encryption. In [20], Li et al. considered a practical scenario in which the scheme provides privacy in a mutually untrusting multidata owner outsourced model but did not consider hiding the data access pattern. In [17], Songhori et al. presented a method to generate a compact PP*k*NN using garbled circuit and implemented it, but they did not consider multiple data owners. In [18], Zhu et al. proposed an efficient PP*k*NN scheme providing data privacy, key confidentiality, input query privacy, and query controllability using combination of random matrix transformation, random permutation, additively homomorphic encryption, and dimension extension. However, the scheme does not consider mutually untrusting multidata owner outsourced model. The work of [19] provided privacy of data owner and inquirer by constructing oblivious kd-tree and oblivious bounded priority queue, but it does not consider multiple data owners. The work of [21] provided data privacy, input query privacy, PP*k*NN result privacy, and hiding access pattern and considered multiple data owners. But, it allows for data owners to send horizontally partitioned data rather than vertically partitioned data.

### 6.2. Privacy Preserving Top-*k* Protocols

In [38, 39], Vaidya and Clifton researched the problem to find the top-*k* elements over vertically partitioned private data using MPC to extend Fargin's algorithm [40]. In [41], Aggarwal et al. designed the protocol to find the *k*-th smallest element over horizontally partitioned data using a binary search. Specifically, the protocol proposes the median of an expected range as a candidate element and counts the number of data smaller than the candidate element over every binary search round. When the count is more than *k*, the range bigger than the candidate element is removed from the expected range since the *k*-th smallest element is smaller than the candidate element, and vice versa. The above process is repeated until the count is same as *k*. However, it is carried out over horizontally partitioned data. In [22], Burkhart et al. proposed the PPTKS protocol to find the top-*k* values over an aggregated key-value list, where the basic idea is the same binary search of [41]. However, the difference from [41] is that PPTKS uses a hash function, and hence it is efficient for sparsely distributed data such as an IP address. However, since PPTKS outputs a probabilistic result because of the hash function, it is unsuitable for application to the e-health handling of sensitive health information. In [42], Jonsson et al. proposed a privacy preserving sorting protocol with MPC in a sorting network and a privacy preserving top-*k* protocol using the sorting protocol, but the running time of their top-*k* protocol is longer than that of the proposed PE-FTK.

## 7. Conclusion

In this paper, we proposed PP*k*NN suitable for medical diagnosis using MPC based on secret sharing in multiple medical data owner environment. The proposed PP*k*NN provides the privacy of medical diagnosis dataset outsourced from multiple data owners, a symptom of patient inquirer and diagnosis result as *k*NN result and hides the data access pattern. As a building block of the proposed PP*k*NN, we proposed the protocol to find *k* data with the highest similarity, which is more efficient than the previous work [22] since it reduces the expensive MPC comparison operation. Furthermore, as the number of data, the length of data, or *k* increase, the number of rounds of PE-FTK does not increase. The proposed PE-FTK returns deterministic results in comparison with the previous work [22]. We expect that researchers construct the privacy preserving and efficient protocols for other data mining techniques other than *k*NN to apply MPC.

## Figures and Tables

**Algorithm 1:**
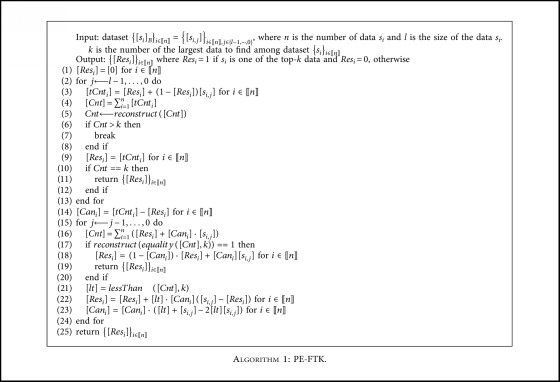
PE-FTK.

**Algorithm 2:**
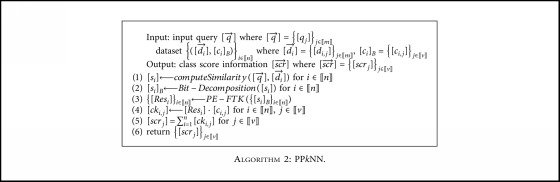
: PP*k*NN.

**Table 1:** Functionality comparison with related works.

Functionality	[20]	[21]	Our study
Privacy of dataset	O	O	O
Privacy of input query	O	O	O
Privacy of *k*NN result	X	O	O
Privacy of data access pattern	X	O	O
Robustness for collusion attack	O	X	O

**Table 2:** Notations for MPC operations.

Syntax	Output
[a] + [b], [a] + b	[a+b]
[a]−[b], [a] − b	[a−b]
[a]∗[b], [a]∗b	[a∗b]
*lessThan* ([a], b)	[1] if a < b, and
	[0] otherwise
*equality* ([a], b)	[1] if a == b, and
	[0] otherwise
*reconstruct*([a])	a

**Table 3:** Example of PE-FTK (dataset {16, 12, 11, 10, 9} and *k* = 3).

Data	Data in binary
16	1 0 0 0 0
12	0 1 1 0 0
11	0 1 0 1 1
10	0 1 0 1 0
9	0 1 0 0 1

**Table 4:** 

Bit-round	(*Cnt*, *k*)	Step	Result set	Candidate set
1	1 < 3	2-3	{16}	
2	5 > 3	2-1	{16}	{12, 11, 10, 9}
3	2 < 3	5-3	{16, 12}	{11, 10, 9}
4	4 > 3	5-2	{16, 12}	{11, 10}
5	3 == 3	5-1	{16, 12, 11}	

**Table 5:** Running time of PE-FTK (seconds).

Operation	Average running time for one round	Total running time
[22]	18.3	132.8–332.7
Our study	12	106.7–118.8

**Table 6:** Round complexity and communication complexity of PE-FTK (*l* is the data size, *n* is the number of data, and *α* is the execution count of part 1).

Operation	Comparison		Equality		Result
	Round	Communication	Round	Communication	
[22]	2*l*	(*n* + 1)*l*	*l*	*l*	Probabilistic
Our study	*l* − *α*	*l − α*	*l − α*	*l − α*	Deterministic

## Data Availability

The data used to support the findings of this study are included within the article.
